# A clinical investigation comparing the efficacy of composite resin vs. resin-infiltrated ceramic CAD/CAM onlays for restoring permanent first molars affected by severe molar-incisor hypomineralization

**DOI:** 10.3389/fdmed.2025.1696662

**Published:** 2025-10-27

**Authors:** Shuya Zhao, Yujia Ren, Yuling Yin, Xianghui Xing

**Affiliations:** Department of Pediatric Dentistry, Nanjing Stomatological Hospital, Affiliated Hospital of Medical School, Nanjing University, Nanjing, China

**Keywords:** resin-infiltrated ceramic CAD/CAM onlays, composite resin restorations, molar-incisor hypomineralization, permanent first molars, USPHS

## Abstract

**Background:**

The restoration of severely affected first permanent molars in patients with molar-incisor hypomineralization (MIH) presents significant clinical challenges yet remains essential.

**Aim:**

The purpose of this randomized controlled trial is to evaluate and compare the clinical performance of the traditional composite resin restorations and the resin-infiltrated ceramic Computer-Aided Design/Computer-Aided Manufacturing onlays on permanent first molars affected with severe MIH.

**Methods:**

Eighty permanent first molars affected with severe MIH in 79 participants (mean age, 8.6 years) were randomly allocated to the two treatment groups: the composite resin restoration (CR Group, *n* = 40) or the resin-infiltrated ceramic CAD/CAM onlays (Lava Ultimate, LU Group, *n* = 40). After complete removal of MIH-affected tissue and tooth preparation, the CR group received direct composite fillings, while the LU group received adhesively cemented CAD/CAM onlays. Follow-up assessments were conducted at 6 months (*n* = 80 restorations), 12 months (*n* = 78), and 24 months (*n* = 68). The restorations were evaluated according to the modified United States Public Health Service (USPHS) criteria with grades Alpha to Delta. Rating with Charlie or Delta was defined as clinical failure. Outcomes assessed included retention, marginal adaptation, secondary caries, color match, and surface roughness. Data were statistically analyzed using Chi-square test, Fisher's exact test, Kaplan–Meier and Log-rank test.

**Results:**

Cumulative survival rates at 6-month (CR Group: 100%; LU Group: 100%), 12-month (CR Group: 89.7%; LU Group: 94.9%), and 24-month (CR Group: 73.4%; LU Group: 86.7%) intervals showed no statistically significant difference (Log-rank test: *χ*^2^ = 1.9, *p* = 0.17). However, the LU Group demonstrated superior performance in clinical indicators including anatomic form maintenance (*p* = 0.02) and recurrent caries incidence (*p* = 0.04) than the CR Group.

**Conclusion:**

Both the composite resin restorations and the resin-infiltrated ceramic CAD/CAM onlays can offer relatively reliable restorative approaches for first molars severely affected by Molar Incisor Hypomineralization (MIH). Adhesive bonding procedures need special attention and a regular maintenance is suggested. A long follow-up is proposed to draw further conclusions.

## Introduction

Molar-incisor hypomineralization (MIH) is a qualitative enamel defect characterized by hypomineralization, with a reported global prevalence ranging from 3.6% to 25% (1, 2). The hypomineralized enamel presents with a variable degree of alteration, ranging from well-demarcated opacities to severe enamel breakdown. Extensive enamel breakdown is strongly correlated with both hypersensitivity and an increased risk of caries. of caries. Severe MIH affected-permanent molars often exhibit fragile enamel, susceptibility to demineralization, fractures, and secondary caries, leading to substantial loss of tooth structure and significant impairment of masticatory function and patient's quality of life (3, 4). Although considerable studies have been conducted on the management of MIH-affected teeth, evidence regarding the definitive restorative method of severely MIH-affected permanent first molars (PFMs) is still lacking (5).

Previous clinical trials suggested that composite resin restorations had shown some desirable positive outcomes in mild and moderate defects of MIH (6–8). Stainless-steel crowns (SSCs) are used to repair severely damaged primary teeth, which is attributable to their ease of use and excellent mechanical properties. These full-coverage crowns have a number of additional advantages in MIH-affected teeth where multiple surfaces are involved, including low cost, immediate alleviation from hypersensitivity, and simple tooth preparation that can be placed in one visit (4, 9, 10). However, SSCs are usually used as an interim option due to their metallic appearance, which may be aesthetically unappealing to the children or their parents. Furthermore, one study reported the adverse effects of SSCs on the periodontium as an increased periodontal pocket depth was observed in the short term (10). Other full-coverage crowns like all-ceramic crowns are in some ways not strongly recommended for young PFMs as they necessitate more extensive tooth preparation (11).

Given the dual need for durability and conservation, onlays may be a potential restorative choice for severely MIH-affected molars. Over the last decades, various materials including metallic, ceramic, and composite resin for onlays have been explored. Onlays produced using ceramics are an aesthetic alternative to metal onlays and have shown acceptable long-term results (12). They have a higher wear resistance than resin materials, but cause more wear to the opposing teeth. At the same time, due to the inherent brittleness of ceramics, fractures at stress-concentration sites were commonly observed (13). The selection of restorative material needs to be cautiously considered when we are dealing with young permanent teeth. Lava Ultimate (3M ESPE) is one kind of resin-infiltrated ceramic material that consists of a polymeric composite framework with embedded ceramic particles (14). They provide a supposed cushion effect at load-bearing sites compared to the more brittle ceramics so that fewer fractures result, and there is presumably less damage to the wear of the opposing teeth (15, 16). Computer-aided design and computer-aided manufacturing (CAD/CAM) systems are widely employed in clinics for teeth restoration in adult populations. CAD/CAM onlays, utilizing high-precision digital impressions and milling technologies, achieve optimal adaptation between the restoration and residual tooth structure, significantly enhancing marginal sealing and long-term prognosis.

Current research on treatment options for severe MIH-affected permanent molars predominantly focuses on short-term outcomes of individual techniques (12, 17, 18), lacking systematic comparisons between conventional composite resin restorations and resin-infiltrated ceramic CAD/CAM onlays in terms of biomechanical properties, clinical survival rates, and cost-effectiveness (19). Therefore, the aim of this randomized controlled trial was to explore the core advantages and potential challenges of these two techniques when applied to MIH-affected molars, providing evidence-based insights for clinical decision-making and advancing MIH treatment toward a precision medicine paradigm that prioritizes both functional rehabilitation and biomechanical compatibility.The null hypothesis was that there would be no significant difference in the survival rate or clinical performance scores between the two restorative approaches at the 24-month follow-up.

## Material and methods

### Recruitment

This randomized controlled trial received approval from the Institutional Ethical Board of Nanjing Stomatological Hospital (NJSH-2021NL-062) and was conducted in accordance with the Declaration of Helsinki. Prior to enrollment, written informed consent was obtained from the parents or guardians of all participants.

Between January and August 2021, 79 pediatric patients (mean age: 8.6 years, range: 7–11) with a total of 80 severely affected permanent first molars (PFMs) were recruited. Children's cooperative behavior was assessed prior to enrollment using the Frankl Behavior Rating Scale (FBRS) (20). Only children rated as 3 (positive) or 4 (definitely positive) were included; for those with dental anxiety, non-pharmacological behavior management strategies (e.g., tell-show-do) were uniformly applied (20). The sample size was based on the difference in restoration survival rates reported in previous literature (12), with a power of 0.8, a significance level of 0.05, and an anticipated attrition rate of 20%. This resulted in a target of 40 restorations per group (total *N* = 80).

### Randomization and allocation concealment

Eligible participants were randomly allocated into two equal groups (*n* = 40 per group): the Composite Resin (CR) group or the Lava Ultimate (LU) onlay group. The randomization sequence was generated by an independent statistician using a computer-based random number generator. To prevent selection bias, the allocation sequence was concealed using sequentially numbered, opaque, sealed envelopes, which were opened only after the treatment assignment was finalized.

The diagnosis of MIH was based on the European Academy of Paediatric Dentistry (EAPD) 2003 criteria (21) and according to the objective criteria of Oliver et al. (22). MIH-affected molars with a total score of 10–13 were considered severe. No signs of rarefaction or periodontal ligament widening were confirmed on the preoperative periapical radiographs.In children having multiple MIH-affected molars, each molar was considered as an individual unit.

We excluded MIH-affected PFMs with mild isolated defects, including those with subgingival defects. PFMs with a history of lingering spontaneous pain or the periapical pathology in radiographic exams were also excluded. Children who suffered from any debilitating systemic disorder were not included.

### Clinical technique

**Figure 1:**
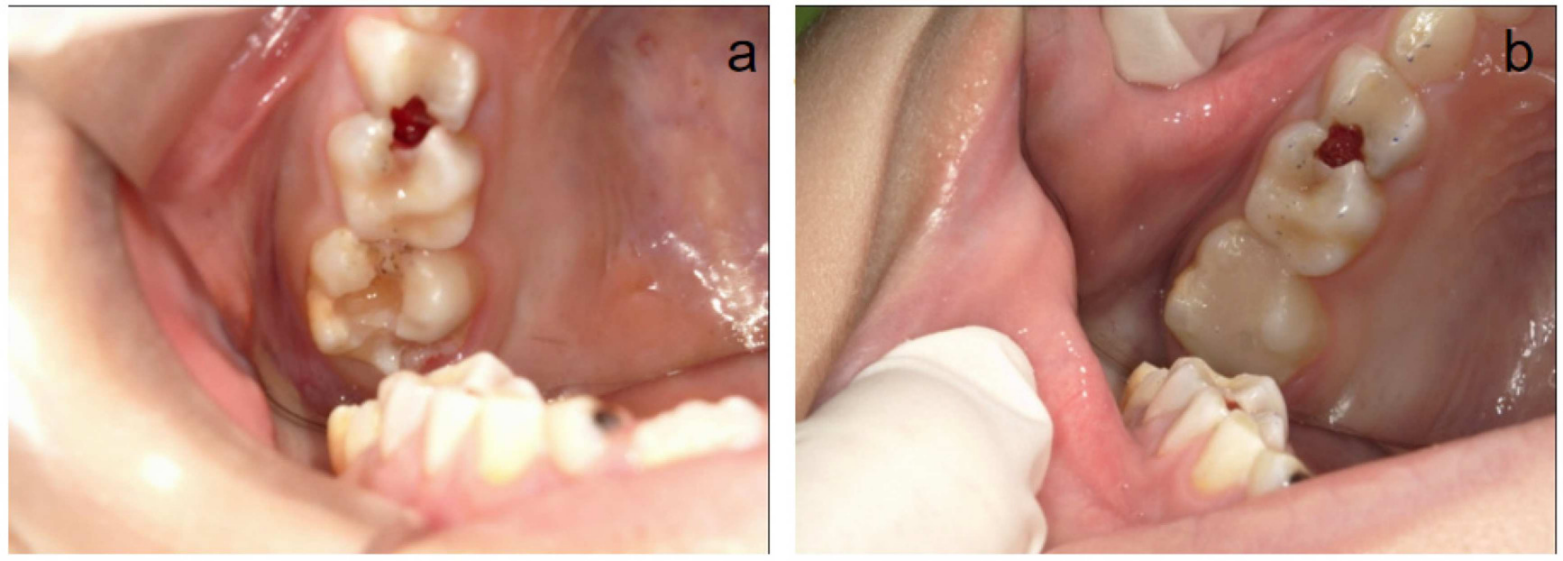
The restoration process of tooth 16 using composite resin. **(a,b)** Preoperative and postoperative situation of an MIH-affected tooth 16.

The entire clinical procedure was carried out by a single trained pediatric dentist. Before tooth preparation, local anesthesia was administered using 2% lidocaine (Lox 2%, Neon laboratories) with 1:80,000 adrenaline for nerve blocks, and the tooth was isolated with a rubber dam. The efficacy of anesthesia was confirmed by the absence of a painful response to initial tooth preparation. In cases of inadequate anesthesia, supplemental techniques (e.g., intraligamentary) were employed to ensure patient comfort, following protocols adapted for hypersensitive MIH teeth. Hypomineralized enamel at the margins of the cavity was selectively removed using high-speed, water-cooled diamond fissure burs. Carious dentin tissue was removed with a steel round bur using a low speed with micromotor. To ensure complete removal of MIH-affected tissue while preserving healthy enamel, a caries detector dye (Sable™ Seek, Ultradent) was applied according to the manufacturer's instructions. Stained areas were re-prepared until no dye retention was observed. A pulp capping material (Calcimol LC, VOCO GmbH, Cuxhaven, Germany) was applied in cases where the remaining dentine thickness was estimated to be <2 mm. Figure 1 shows the restoration of MIH-affected PFMs using composite resin.

In the CR Group, the teeth were etched using 37% phosphoric acid (Scotchbond™ Multi Purpose Etchant 3M ESPE) for 30 s, thoroughly rinsed with water, and dried with an air syringe. Subsequently, the adhesive (Clearfil™ SE Bond, Kuraray Noritake Dental) was applied to the entire surface of the remaining tooth structure, gently air-thinned, and light-cured for 20 s. Finally, the teeth form were restored using layered composite resin (Filtek TM Z350XT, A2 shade, 3M ESPE St. Paul, USA) filling. Articulating papers were employed to check for occlusal high spots, followed by form adjustment of the restorations using bud-shaped finishing burs, and polishing with rubber cups.

In the LU Group, after placing the base material, an intraoral scanner (iTero Element, Align Technologies, San Jose, California, USA) was used to scan the preparation, antagonist teeth, and buccal bite registration. The prefabricated blocks of Lava Ultimate were designed and milled by the clinician at standard milling speed. Diamond fissure burs were then used to adjust the restorations.

All Lava Ultimate restorations were cleaned with 96% isopropyl alcohol. The inner surfaces were subjected to sandblasting treatment with 50 μm alumina particles at an air pressure of 30 PSI, then they were applied with silane for 60 s. Finishing and polishing were conducted with SofLex discs (3M ESPE, Seefeld, Germany).

At the cementation stage, the teeth were etched using 37% phosphoric acid (Scotchbond™ Multi Purpose Etchant 3M ESPE) for 30 s and applied the entire surfaces of restorations with an adhesive (Tetric N-Bond; Ivoclar Vivadent). Dual cure resin cement (Rely XTM Unicem 2 ClickerTM 100, 3M ESPE) was used for final cementation. Figuress 2, 3 show the restorations of MIH-affected PFMs using Lava Ultimate onlays and their follow-up at 24 months.

**Figure 2:**
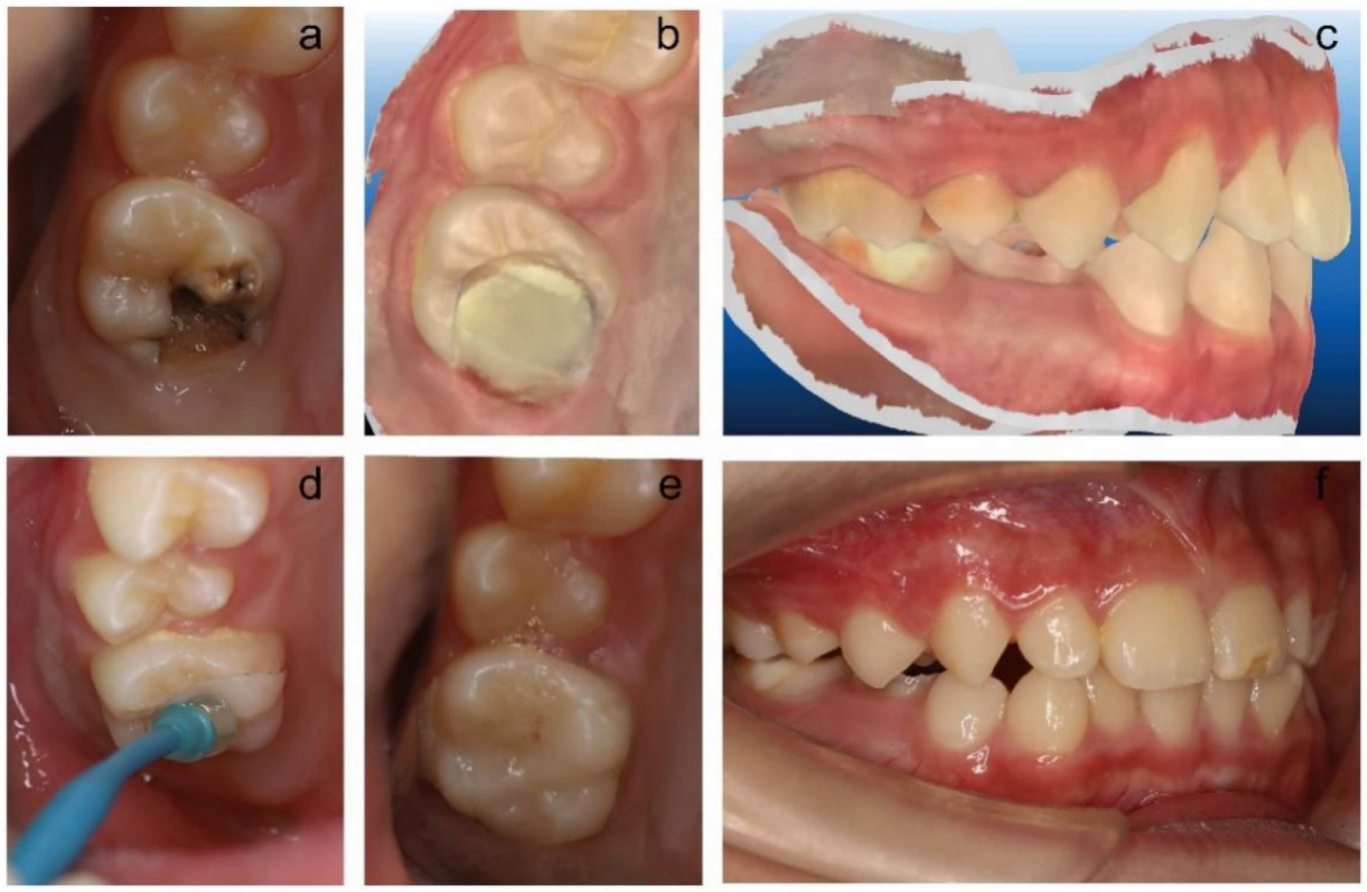
The restoration process of tooth 16 using resin-infiltrated ceramic CAD/CAM onlays. **(a)** Preoperative situation of an MIH-affected tooth 16 with caries. **(b)** A scan of the occlusal surface of 16 after tooth preparation. **(c)** A scan of occlusal contacts of normal occlusion in the intercuspal position. **(d)** Cementation of restoration. **(e)** Examination of margin and proximal contact. **(f)** Examination of occlusion.

**Figure 3:**
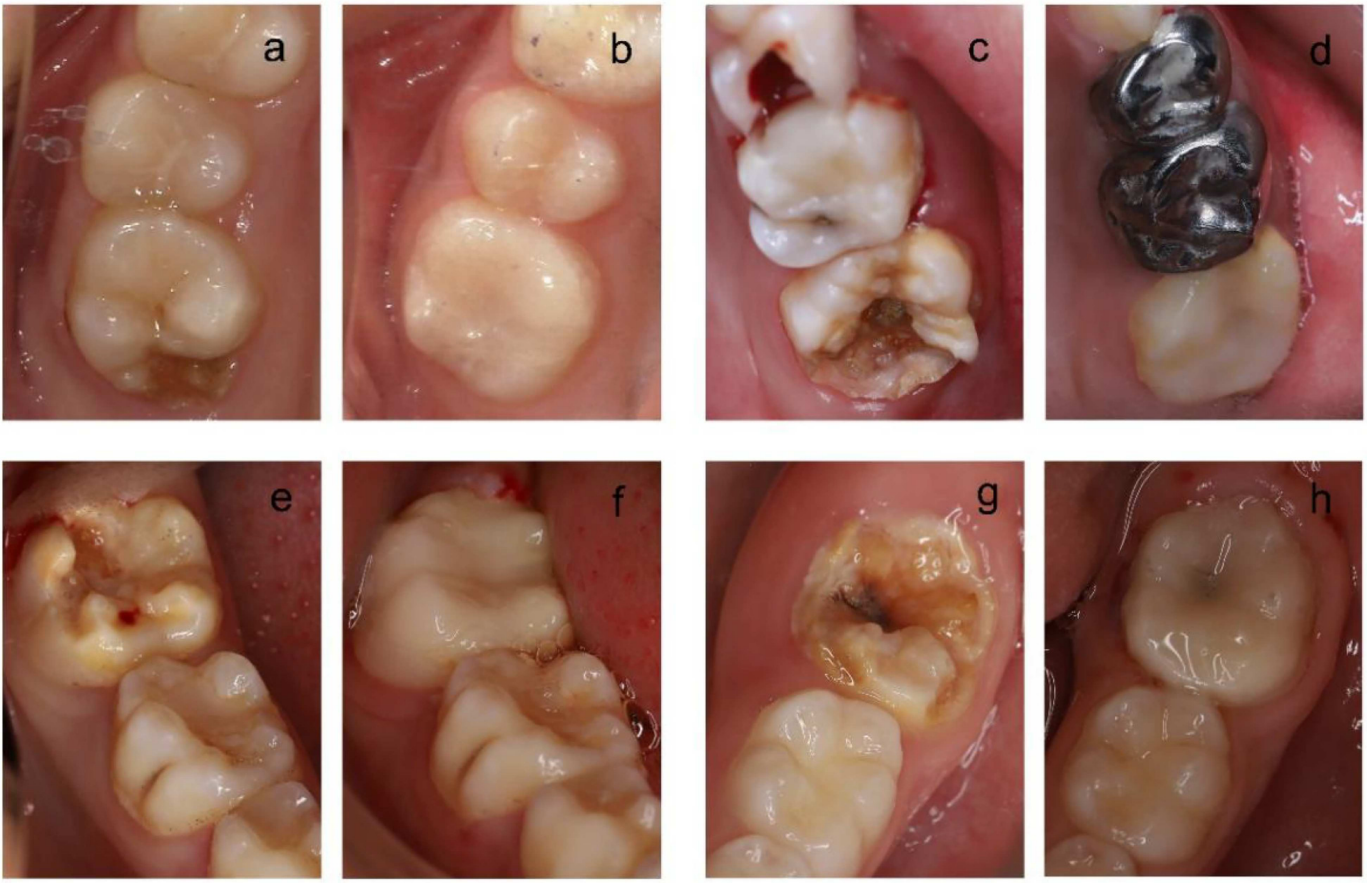
The restoration of teeth using resin-infiltrated ceramic CAD/CAM onlays. **(a,b)** Preoperative and postoperative (24 months post-op) situation of an MIH-affected tooth 16. **(c,d)** Preoperative and postoperative (24 months post-op) situation of an MIH-affected tooth 26. **(e,f)** Preoperative and postoperative (24 months post-op) situation of an MIH-affected tooth 36. **(g,h)** Preoperative and postoperative (24 months post-op) situation of an MIH-affected tooth 46.

### Evaluation

The participants were evaluated at baseline (immediately after restoration placement) and at 6, 12, and 24 months after restoration for the consequent development of any clinical symptoms. Two independent examiners assessed the restorations and teeth using the modified United States Public Health Services (USPHS) Criteria calibrated before the baseline assessment (23). Although examiner blinding was not feasible due to the morphological differences between the two types of restorations, potential bias was mitigated by strict adherence to the objective the modified USPHS criteria. Restorations were defined as survival when they were clinically acceptable in terms of functional, biological, and esthetic evaluation categories (Alpha or Bravo scores). Failure was considered whenever a restoration was graded as Charlie or Delta. The restoration graded as Charlie was considered unacceptable but still repairable, while the restoration graded as Delta was considered a failure with the immediate need for replacement, which was defined by debonding (loss of retention) or any esthetic failure and mechanical complications like a severe fracture or chipping of ceramic that were clinically unacceptable. The treated teeth were then examined clinically. Asymptomatic teeth without radiographic and clinical evidence of periapical pathology were considered success. Teeth with spontaneous pain or periapical lesions which required endodontic intervention were listed as failed.

### Statistical analysis

Data analysis was performed using IBM SPSS Statistics (Inc., Chicago, version 22.0). Chi-square test and Fisher's exact test were employed to compare the differences in clinical scores between the two groups. Survival analysis of the restorations in both groups was performed using the Kaplan–Meier method and Log-rank test. All tests were two-tailed and performed at a significance level of *α* = 0.05. The statistical analysis plan was reviewed and approved by an independent statistician to ensure the appropriateness of the chosen methods.

## Results

The study included 80 first molars diagnosed with MIH (Molar Incisor Hypomineralization) from 79 pediatric participants aged 7–11 years. This cohort consisted of 34 boys and 45 girls. Table 1 presented the distribution of molars included in the analysis.

**Table 1:** Molars distribution.

Molars distribution	Boys	Girls	Total
Maxillary molars	15 (18.75%)	21 (26.25%)	36 (45%)
Mandibular molars	19 (23.75%)	25 (31.25%)	44 (55%)
Total	34 (42.5%)	46 (57.5%)	80 (100%)

### Restoration failures

In the CR Group, ten restorations were rated as clinical failures until the final follow-up. At the 6-month follow-up, there was no case lost and all restorations remained functional. At the 12-month follow-up, one participant was lost to follow-up and four restorations were assessed as clinical failures: one exhibited complete debonding at 11 months, one demonstrated poor marginal adaptation, one showed compromised anatomic form, and one developed severe secondary caries. These five cases were excluded from the next clinical evaluation.

At the 24-month evaluation, two participants were lost to follow-up and six additional failures occurred: two restorations debonded, one had poor marginal adaptation, one displayed compromised anatomical form, and two developed severe secondary caries. For teeth with failed resin restorations, carious tissue was removed under rubber dam isolation, followed by re-restoration with composite resin.

In the LU Group, five restorations were rated as clinical failures until the final follow-up. At the 6-month follow-up, there was no case lost and all restorations remained functional. At the 12-month follow-up, one participant was lost to follow-up and the first two failures occurred. One restoration failed due to debonding which was loose but did not get dislodged from the tooth. This participant reported a history of bruxism and restoration with SSC was adopted finally. The second failure was found in a maxillary left molar, with a minor chip at the buccal margin of the restoration. The restoration was scored C and repaired with direct composite resin under rubber dam isolation. These three cases were excluded from the next clinical evaluation.

At the 24-month evaluation, two participants were lost to follow-up and three restorations failed. Two onlays got dislodged from the teeth. No cracks within the tooth could be detected. Debonding of the restoration occurred at 14 and 19 months after insertion respectively. The original restorations were lost and new onlays were fabricated with Lava Ultimate and iTero. One failure was identified as a minor fracture of the distal cusp of the mandibular first molar. A new onlay was fabricated with Lava Ultimate and iTero. A slight chip at the lingual-mesial margin of the restoration in a maxillary left first molar was also found and scored C. The fracture was repaired with direct composite resin under rubber dam isolation.

**Figure 4:**
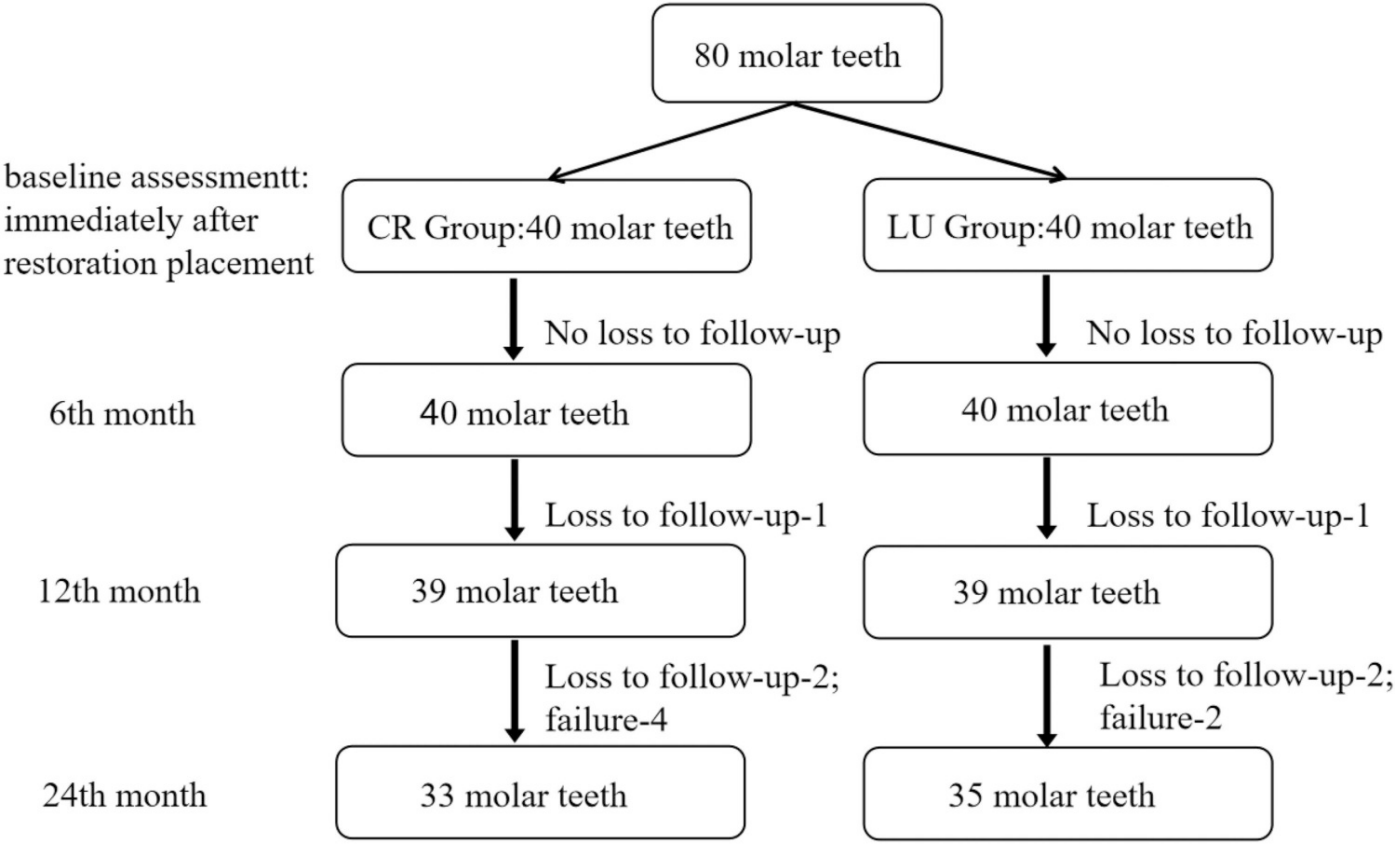
The flow diagram of participants during the two-year follow-up period.

At the 6-, 12-, and 24-month follow-ups, the follow-up rates were 100%, 97.5%, and 94.4%, while the drop-out rates were 0%, 2.5%, and 5.6%, respectively. The flow diagram of participants during 2 years is presented in Figure 4.

### Evaluation of dental data

Chi-square test and Fisher's exact tests demonstrated no statistically significant differences (*p* > 0.05) between the CR and the LU groups in restoration retention rates, marginal adaptation, color match, and surface roughness over the 24-month follow-up period. However, at the 24-month follow-up, the LU Group demonstrated superior restorative outcomes compared to the CR Group in terms of anatomic form (*p* = 0.02) and resistance to secondary caries (*p* = 0.04). Detailed results for clinical evaluation at 6M, 12M, and 24M follow-up are summarized in Table 2.

**Table 2:** Clinical evaluation of two groups at 6,12and 24 months.

Evaluation parameter	Baseline			6th month			12th month			24th month		
	CR Group N (%)	LU Group N (%)	*p* value	CR Group N (%)	LU Group N (%)	*p* value	CR Group N (%)	LU Group N (%)	*p* value	CR Group N (%)	LU Group N (%)	*p* value
Retention												
Alpha	40 (100)	40 (100)	—	40 (100)	40 (100)	—	38 (97.4)	38 (97.4)	—	32 (97)	33 (94.3)	>0.99
Charlie	0	0		0	0		1 (2.6)	1 (2.6)		1 (3)	2 (5.7)	
Marginal adaptation												
Alpha	39 (97.5)	40 (100)	0.31	37 (92.5)	39 (97.5)	0.62	33 (86.8)	35 (92.1)	0.71	23 (71.9)	27 (81.8)	0.46
Bravo	1 (2.5)	0		3 (7.5)	1 (2.5)		4 (10.5)	3 (7.9)		8 (25)	6 (18.2)	
Charlie	0	0		0	0		1 (2.6)	0		1 (3.1)	0	
Delta	0	0		0	0		0	0		0	0	
Anatomic form												
Alpha	39 (97.5)	40 (100)	0.31	38 (95)	40 (100)	0.49	32 (84.2)	36 (94.7)	0.2	21 (65.6)	30 (90.9)	0.02*
Bravo	1 (2.5)	0		2 (5)	0		5 (13.2)	1 (2.6)		10 (31.3)	2 (6.1)	
Charlie	0	0		0	0		1 (2.6)	1 (2.6)		1 (3.1)	1 (3)	
Delta	0	0		0	0		0	0		0	0	
Secondary caries												
Alpha	40 (100)	40 (100)	—	37 (92.5)	39 (97.5)	0.62	33 (86.8)	36 (94.7)	0.43	21 (65.6)	30 (90.9)	0.04*
Bravo	0	0		3 (7.5)	1 (2.5)		4 (10.5)	2 (5.3)		8 (25)	3 (9.1)	
Charlie	0	0		0	0		1 (2.6)	0		2 (6.3)	0	
Delta	0	0		0	0		0	0		1 (3.1)	0	
Color match												
Alpha	38 (95)	40 (100)	0.49	39 (97.5)	39 (97.5)	—	33 (86.8)	35 (92.1)	0.71	24 (75)	28 (84.8)	0.32
Bravo	2 (5)	0		1 (2.5)	1 (2.5)		5 (13.2)	3 (7.9)		8 (25)	5 (15.2)	
Charlie	0	0		0	0		0	0		0	0	
Surface roughness												
Alfa	38 (95)	39 (97.5)	0.56	36 (90)	38 (95)	0.68	32 (84.2)	35 (92.1)	0.48	23 (71.9)	27 (81.8)	0.34
Bravo	2 (5)	1 (2.5)		4 (10)	2 (5)		6 (15.8)	3 (7.9)		9 (28.1)	6 (18.2)	
Charlie	0	0		0	0		0	0		0	0	
Delta	0	0		0	0		0	0		0	0	

The modified USPHS criteria ranked from best (Alpha) to worst (Delta). Fisher's exact test indicated that at the 24-month follow-up post-restoration, the LU Group demonstrated statistically significant superiority over the CR group regarding anatomical form maintenance (*p* = 0.02) and secondary caries prevention (*p* = 0.04). For other clinical scores, there were no statistically significant differences between the two groups (*p* > 0.05).

*p-value <0.05.

**Figure 5:**
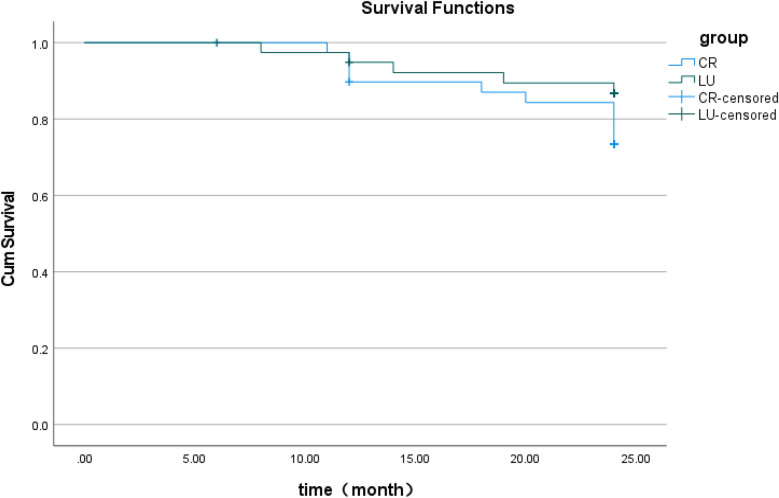
Kaplan–meier survival analysis of the CR group and LU group. Kaplan–Meier analysis and Log-rank test over a 24-month period demonstrated that the LU Group exhibited a marginally higher survival rate of restorations compared to the CR Group, but this difference was not statistically significant (*p* = 0.17).

Survival analyses based on categorical factors were described with Kaplan–Meier survival plots. Log-rank test demonstrated comparable cumulative survival rates between restoration groups. Cumulative survival rates at 6-month (the CR Group: 100%; the LU Group: 100%), 12-month (the CR Group: 89.7%; the LU Group: 94.9%), and 24-month (the CR Group: 73.4%; the LU Group: 86.7%) intervals showed no statistically significant difference (Log-rank test: *χ*^2^ = 1.9, *p* = 0.17). All the remaining 59 restored teeth retained their vitality after clinical examination and radiographic assessment. The survival curve is presented in Figure 5.

## Discussion

This study compared direct composite resin restorations with resin-infiltrated ceramic CAD/CAM onlay restorations for first molars severely affected by Molar Incisor Hypomineralization (MIH) through a 24-month randomized controlled trial. The 2-year restoration success rates were 73.4% (composite resin/CR) and 86.7% (resin-infiltrated ceramic/LU), with no statistically significant difference (*p* > 0.05), indicating comparable short-term reliability for both restorative techniques. Thus, the null hypothesis—that there would be no significant difference in survival rates between the two restorative approaches—was accepted.

However, in clinical indicators such as anatomic form maintenance and recurrent caries incidence, restorations in the LU Group demonstrated significant advantages (*p* < 0.05). At the follow-up endpoint, the proportion of Lava Ultimate restorations achieving Alpha-level anatomic form integrity scores (the modified USPHS criteria) was substantially higher than that in the composite resin group (90.9% vs. 65.6%, *p* = 0.013). Additionally, the incidence of recurrent caries was significantly greater in the CR Group than in the LU Group (34.4% vs. 9.1%, *p* = 0.04).Therefore, the null hypothesis is rejected for these specific clinical parameters.

At the end of follow-up, the LU restorations exhibited significantly superior anatomic form scores compared to the CR Group. This difference may be attributed to the elastic modulus of Lava Ultimate (21.40 ± 1.56 GPa) closely approximates that of natural dentin (25 GPa), whereas composite resin (13.25 GPa) displays a relatively lower modulus and consequently greater susceptibility to plastic deformation under prolonged stress (24, 25). Furthermore, Lava Ultimate incorporates an 80% nano-zirconia/silica filler framework that establishes a rigid skeleton, contrasting with the 57% filler content (silica nanofillers and zirconia/silica nanoclusters) in the composite resin (Filtek TM Z350XT, A2 shade, 3M ESPE St. Paul, USA) (25). Compared to resin-based materials, the LU Group demonstrated enhanced wear resistance. Given that MIH-affected teeth exhibit uneven enamel mineralization that accelerates occlusal surface wear, the high filler load of Lava Ultimate effectively maintains the functional occlusal plane while reducing pathological wear on dental surfaces.

The significantly higher recurrent caries incidence in the CR Group may be explained by several factors. Primarily, during direct composite resin restorations, polymerization shrinkage of resin monomers (typically 1.5%–3%) generates contraction stresses (26). These stresses can induce microleakage at the resin-dentin interface, creating pathways for bacterial infiltration. In contrast, as a pre-polymerized CAD/CAM block material, Lava Ultimate exhibits negligible polymerization shrinkage, substantially mitigating microleakage risks.

Additionally, research by Feilzer et al. demonstrates that composite resins undergo hygroscopic expansion when exposed to the moist oral environment (27, 28). While this water absorption may temporarily compensate for microleakage (28), repeated wet-dry cycles can induce dimensional instability in the material, ultimately may accelerate marginal failure.

Furthermore, three out of five restoration failures in onlays were associated with debonding in this study. These observations might refer to the relatively low bonding strength of particle-filled nanoceramic resin restorations such as Lava Ultimate compared with conventional ceramics. Frankenberger et al. found that the highest micro tensile bond strength for Lava Ultimate restorations was 17.9 ± 4.5 MPa *in vitro*, while the bond strength of Lithium-disilicate ceramics were found to be 26.3 ± 7.7 MPa (16). Furthermore, some scholars argued that the restoration material and luting resin composite might be the weak spot of adhesive process, if the bonding protocol is not meticulously followed, it seems to be more susceptible to bonding failure (29). In addition, for severely MIH-affected molars, adhesive failure may result from the weaker bond strength between the hypo-mineralized tooth structure (which persists at an ultra-structural level) and the resin cement (26). Pre-treatment with 5.2% sodium hypochlorite for MIH-affected teeth was used in some trials to enhance retention of restorations, but no results thus far can definitely show that this technique improves the success of retention substantially (5). Consequently, future research on bonding agents for resin-infiltrated CAD/CAM ceramics represents a promising avenue for scientific exploration.

While the present study provides valuable evidence supporting the use of both restorative strategies for severe MIH, several important clinical considerations and limitations must be acknowledged to contextualize the findings. First, the generalizability of our results is influenced by the specific patient cohort. All participants were required to exhibit positive cooperative behavior (rated as FrankI 3 or 4) to tolerate the potentially longer chair time associated with the CAD/CAM workflow (20). This selection criterion underscores a key practical advantage of direct composite resin restorations in real-world clinical settings. For children with significant dental anxiety or limited cooperation, the shorter, single-visit nature of direct composite placement may represent a more feasible and pragmatic choice, despite the observed superior performance of onlays in certain biomechanical indicators (20, 30).

Second, the challenge of achieving profound local anesthesia in symptomatic MIH molars is well-documented, often attributed to chronic inflammation and altered innervation of the pulp (30). Although our randomization protocol and standardized anesthesia techniques aimed to mitigate this potential bias, the less invasive and faster composite resin procedure may inherently confer an advantage in situations where anesthesia is difficult to secure. Future studies could stratify randomization based on preoperative pulp symptomatology to further elucidate this interaction.

Finally, regarding tooth preparation, a potential confounding factor when comparing direct and indirect techniques is the extent of tissue removal. One might hypothesize that more extensive preparation for onlays might extend into healthier enamel. However, in this study, a standardized, tissue-driven protocol using caries detector dye was employed to ensure all cavity margins terminated in sound, dye-free enamel (31). This method aimed to minimize bias by ensuring that the quality of the adhesive substrate at the margins was comparable between groups. Therefore, the superior marginal adaptation and caries resistance observed in the LU group are more likely attributable to the material properties (e.g., reduced polymerization stress, higher wear resistance) and precision fit of the CAD/CAM onlays rather than to a systematically healthier enamel margin.

Furthermore, this study is limited by its relatively short follow-up period (2 years), during which the long-term wear resistance advantage of Lava Ultimate may become more pronounced beyond 5 years. Future investigations should prioritize exploring novel bonding systems adapted for MIH-affected teeth and evaluate the potential of AI-assisted and digital workflows to optimize the marginal fit and overall longevity of such restorations.

## Conclusion

This two-year follow-up study confirms that for severely MIH-affected first permanent molars, the restoration success rates were 73.4% for composite resin and 86.7% for resin-infiltrated CAD/CAM ceramic onlays (Lava Ultimate), with no statistically significant difference (*p* > 0.05), indicating both techniques provide reliable short-term outcomes. However, the Lava Ultimate group demonstrated superior performance in clinical indicators including anatomic form maintenance and recurrent caries incidence (*p* < 0.05).A long follow-up is proposed to draw further conclusions.

## Data Availability

The original contributions presented in the study are included in the article/Supplementary Material, further inquiries can be directed to the corresponding author.

## References

[1] Weerheijm KL Jälevik B Alaluusua S. Molar-incisor hypomineralisation. Caries Res. (2001) 35(5):390–1. 10.1159/000047479 11641576

[2] Weerheijm KL Mejàre I. Molar incisor hypomineralization: a questionnaire inventory of its occurrence in member countries of the European Academy of Paediatric Dentistry (EAPD). Int J Paediatr Dent. (2003) 13(6):411–6. 10.1046/j.1365-263x.2003.00498.x 14984047

[3] William V Messer LB Burrow MF. Molar incisor hypomineralization: review and recommendations for clinical management. Pediatr Dent. (2006) 28(3):224–32. https://pubmed.ncbi.nlm.nih.gov/16805354/16805354

[4] Fayle SA. Molar incisor hypomineralisation: restorative management. Eur J Paediatr Dent. (2003) 4(3):121–6. https://pubmed.ncbi.nlm.nih.gov/14529331/14529331

[5] Lygidakis NA Garot E Somani C Taylor GD Rouas P Wong FSL. Best clinical practice guidance for clinicians dealing with children presenting with molar-incisor-hypomineralisation (MIH): an updated European Academy of Paediatric Dentistry policy document. Eur Arch Paediatr Dent. (2022) 23(1):3–21. 10.1007/s40368-021-00668-5 34669177 PMC8926988

[6] Lygidakis NA Chaliasou A Siounas G. Evaluation of composite restorations in hypomineralised permanent molars: a four year clinical study. Eur J Paediatr Dent. (2003) 4(3):143–8. https://pubmed.ncbi.nlm.nih.gov/14529336/14529336

[7] Rolim TZC da Costa TRF Wambier LM Chibinski AC Wambier DS da Silva Assunção LR Adhesive restoration of molars affected by molar incisor hypomineralization: a randomized clinical trial. Clin Oral Investig. (2021) 25(3):1513–24. 10.1007/s00784-020-03459-2 32696210

[8] Somani C Taylor GD Garot E Rouas P Lygidakis NA Wong FSL. An update of treatment modalities in children and adolescents with teeth affected by molar incisor hypomineralisation (MIH): a systematic review. Eur Arch Paediatr Dent. (2022) 23(1):39–64. 10.1007/s40368-021-00635-0 34110615 PMC8927013

[9] Croll TP. Restorative options for malformed permanent molars in children. Compend Contin Educ Dent. (2000) 21(8):676–8; 680, 682. https://pubmed.ncbi.nlm.nih.gov/11199658/11199658

[10] Koleventi A Sakellari D Arapostathis KN Kotsanos N. Periodontal impact of preformed metal crowns on permanent molars of children and adolescents: a pilot study. Pediatr Dent. (2018) 40(2):117–21. https://pubmed.ncbi.nlm.nih.gov/29663912/29663912

[11] Linner T Khazaei Y Bücher K Pfisterer J Hickel R Kühnisch J. Comparison of four different treatment strategies in teeth with molar-incisor hypomineralization-related enamel breakdown—a retrospective cohort study. Int J Paediatr Dent. (2020) 30(5):597–606. 10.1111/ipd.12636 32112475

[12] Dhareula A Goyal A Gauba K Bhatia SK Kapur A Bhandari S. A clinical and radiographic investigation comparing the efficacy of cast metal and indirect resin onlays in rehabilitation of permanent first molars affected with severe molar incisor hypomineralisation (MIH): a 36-month randomised controlled clinical trial. Eur Arch Paediatr Dent. (2019) 20(5):489–500. 10.1007/s40368-019-00430-y 30888581

[13] Abdulrahman S Von See Mahm C Talabani R Abdulateef D. Evaluation of the clinical success of four different types of lithium disilicate ceramic restorations: a retrospective study. BMC Oral Health. (2021) 21(1):625. 10.1186/s12903-021-01987-1 34876103 PMC8650461

[14] Oz FD Bolay S Canatan S. A clinical evaluation of resin nanoceramic CEREC omnicam restorations associated with several factors. J Esthet Restor Dent. (2021) 33(4):583–9. 10.1111/jerd.12691 33283974

[15] Kramer N Kunzelmann KH Taschner M Mehl A Garcia-Godoy F Frankenberger R. Antagonist enamel wears more than ceramic onlays. J Dent Res. (2006) 85(12):1097–100. 10.1177/154405910608501206 17122161

[16] Frankenberger R Hartmann VE Krech M Kramer N Reich S Braun A Adhesive luting of new CAD/CAM materials. Int J Comput Dent. (2015) 18(1):9–20. https://pubmed.ncbi.nlm.nih.gov/25911826/25911826

[17] Fütterer J Ebel M Bekes K Klode C Hirsch C. Influence of customized therapy for molar incisor hypomineralization on children’s oral hygiene and quality of life. Clin Exp Dent Res. (2020) 6(1):33–43. 10.1002/cre2.245 32067392 PMC7025992

[18] Baroudi K Ibraheem SN. Assessment of chair-side computer-aided design and computer-aided manufacturing restorations: a review of the literature. J Int Oral Health. (2015) 7(4):96–104. https://pubmed.ncbi.nlm.nih.gov/25954082/25954082 PMC4409808

[19] Davidovich E Dagon S Tamari I Etinger M Mijiritsky E. An innovative treatment approach using digital workflow and CAD-CAM part 2: the restoration of molar incisor hypomineralization in children. Int J Environ Res Public Health. (2020) 17(5):1499. 10.3390/ijerph17051499 32110963 PMC7084897

[20] American Academy of Pediatric Dentistry. Behavior Guidance for the Pediatric Dental Patient. The Reference Manual of Pediatric Dentistry. Chicago, Il.: American Academy of Pediatric Dentistry (2024). p. 358–78.

[21] Weerheijm KL Duggal M Mejare I Papagiannoulis L Koch G Martens LC Judgement criteria for molar incisor hypomineralisation (MIH) in epidemiologic studies: a summary of the European meeting on MIH held in Athens, 2003. Eur J Paediatr Dent. (2003) 4(3):110–3. https://pubmed.ncbi.nlm.nih.gov/14529329/14529329

[22] Oliver K Messer LB Manton DJ Kan K Ng F Olsen C Distribution and severity of molar hypomineralisation: trial of a new severity index. Int J Paediatr Dent. (2014) 24(2):131–51. 10.1111/ipd.12040 23701232

[23] Bayne SC Schmalz G. Reprinting the classic article on USPHS evaluation methods for measuring the clinical research performance of restorative materials. Clin Oral Investig. (2005) 9(4):209–14. 10.1007/s00784-005-0017-0 16421996

[24] Jin C Deng J Pan P Xiong Y Zhu L Gao S. Comparative study on the impact-sliding wear behaviour of CAD/CAM resin-ceramic materials and tooth enamel. Dent Mater. (2023) 39(1):25–40. 10.1016/j.dental.2022.11.010 36456379

[25] Li Q Zhan N Ng T Swain MV Wan B Jian Y The influence of hygroscopic expansion of resin supporting dies on the fracture resistance of ceramic restorations during thermal cycling. Dent Mater. (2024) 40(8):1231–43. 10.1016/j.dental.2024.06.011 38853105

[26] Fagrell TG Dietz W Jalevik B Noren JG. Chemical, mechanical and morphological properties of hypomineralized enamel of permanent first molars. Acta Odontol Scand. (2010) 68(4):215–22. 10.3109/00016351003752395 20392131

[27] Feilzer YJ Feilzer AJ Noack MJ Kleverlaan CJ. Release of contraction stress of dental resin composites by water sorption. Dent Mater. (2024) 40(10):1697–170. 10.1016/j.dental.2024.08.001 39098487

[28] Angeletaki F Gkogkos A Papazoglou E Kloukos D. Direct versus indirect inlay/onlay composite restorations in posterior teeth. A systematic review and meta-analysis. J Dent. (2016) 53:12–21. 10.1016/j.jdent.2016.07.011 27452342

[29] Borgia E Baron R Borgia JL. Quality and survival of direct light-activated composite resin restorations in posterior teeth: a 5- to 20-year retrospective longitudinal study. J Prosthodont. (2019) 28(1):e195–203. 10.1111/jopr.12630 28513897

[30] Mathu-Muju K Wright JT. Diagnosis and treatment of molar incisor hypomineralization. Compend Contin Educ Dent. (2006) 27(11):604–10. quiz 611. https://pubmed.ncbi.nlm.nih.gov/17133930/17133930

[31] Boston DW Graver HT. Histological study of an acid red caries-disclosing dye. Oper Dent. (1989) 14(4):186–92. https://pubmed.ncbi.nlm.nih.gov/2639319/2639319

